# The association between the C-reactive protein-triglyceride-glucose index and cardiovascular diseases: A cohort study using data from the China Health and Retirement Longitudinal Study 2011–2020

**DOI:** 10.1371/journal.pone.0335916

**Published:** 2025-12-04

**Authors:** Xinxin Xu, Jianfeng Liu, Yazhao Sun, Pei Sun, Xiao Yu

**Affiliations:** Department of Cardiology, Cangzhou People’s Hospital, Cangzhou, Hebei, China; No affiliation currently, UNITED KINGDOM OF GREAT BRITAIN AND NORTHERN IRELAND

## Abstract

**Background:**

Inflammation and insulin resistance (IR) are both risk factors for cardiovascular disease (CVD). The C-reactive protein-triglyceride-glucose index (CTI) is a novel biomarker that comprehensively assesses the severity of inflammation and IR. This study investigates the association between CTI and the risk of CVD.

**Methods:**

Data from the China Health and Retirement Longitudinal Study (CHARLS) conducted between 2011 and 2020 were utilized, focusing on individuals aged 45 years and older. The CTI was calculated as 0.412 × Ln (C-reactive protein [CRP, mg/L]) + Ln (triglycerides [TG, mg/dL] × fasting blood glucose [FBG, mg/dL]/ 2). Self-reported CVD events were used as the primary outcome measure. Multivariable Cox regression, restricted cubic splines (RCS) analysis, and subgroup analyses were performed to examine the association between CTI and CVD risk.

**Results:**

This study included 5,642 participants, with a CVD incidence rate of 23.70% (1,337 cases). After adjusting for covariates, each unit increase in the CTI was associated with an 11% increase in the risk of CVD (hazard ratio (HR) = 1.11, 95% confidence interval (CI) = 1.01–1.23). Similar results were observed when CTI was analyzed as a categorical variable (quartiles). RCS analyses revealed a nonlinear relationship between CTI and CVD risk. Subgroup analysis revealed no significant associations in certain subgroups, suggesting that the effect of the association may be heterogeneous.

**Conclusion:**

Higher CTI (whether treated as a continuous or categorical variable) was significantly associated with an increased risk of CVD. A nonlinear relationship between CTI and CVD risk was observed.

## 1 Introduction

According to the World Health Organization (WHO) report, cardiovascular disease (CVD) causes more than 18 million deaths globally each year, accounting for 31% of all deaths worldwide [1]. CVD is one of the leading causes of death from non-communicable diseases globally, particularly in low- and middle-income countries, which account for over 80% of global CVD-related deaths [2]. In these countries, the prevention and management of CVD face numerous challenges, including insufficient healthcare resources and lack of public health awareness [3].

C-reactive protein (CRP), an acute-phase reactant and a marker of inflammation, has been widely used to assess the relationship between chronic inflammation and CVD. Studies have shown that elevated CRP levels are associated with an increased risk of CVD, particularly in patients with acute myocardial infarction, where changes in CRP levels reflect the degree of the inflammatory response [4]. In CVD risk assessment, CRP is considered a valuable predictive biomarker and can be used in combination with other parameters to enhance risk prediction and guide treatment decisions [5].

Insulin resistance (IR) plays a significant role in the development of CVD. IR is not only closely associated with type 2 diabetes mellitus (T2DM) but is also linked to various metabolic abnormalities, all of which are well-established risk factors for CVD. Improving insulin sensitivity is expected to lower CVD risk in T2DM patients, even without relying on blood glucose control [6]. Furthermore, studies have shown that IR is associated with the progression of atherosclerosis, particularly through the reduction of vascular smooth muscle cell survival and the increased expression of the CX3CL1/CX3CR1 axis. IR increases plaque vulnerability by enhancing the C-X3-C motif chemokine ligand 1 (CX3CL1)/ C-X3-C motif chemokine receptor 1 (CX3CR1) axis, which mechanistically links to the decreased survival of vascular smooth muscle cells [7].

The triglyceride-glucose (TyG) index is an easily accessible and reliable marker of IR, with performance comparable to that of gold-standard techniques. Research has demonstrated that the TyG index not only effectively reflects IR but is also closely associated with various metabolic disorders and CVD. First, the TyG index is considered an effective marker for predicting arteriosclerosis. One study found that the TyG index was significantly correlated with the risk of arteriosclerosis in healthy participants without diabetes. Measurements of functional vascular diseases using pulse wave velocity (PWV) showed that individuals with a higher TyG index had higher PWV values, suggesting that the TyG index could serve as a potential predictor of arteriosclerosis [8]. Second, the TyG index plays an important role in CVD risk assessment. Studies have indicated that individuals with a higher TyG index have a significantly increased 10-year risk of CVD, as assessed by the Framingham risk score [9]. Moreover, the TyG index has been used to assess the risk of readmission in heart failure patients. One study found that a lower TyG index was associated with an increased risk of readmission within 6 months in heart failure patients, suggesting that the TyG index could serve as an important prognostic indicator for heart failure patients [10].

The C-reactive protein-triglyceride-glucose index (CTI) is an emerging marker for comprehensively assessing the severity of inflammation and IR. CTI uniquely combines both inflammatory and insulin resistance markers, offering a novel approach for predicting CVD risk by jointly evaluating these two important metabolic factors. Studies have shown that CTI is closely related to various health issues. For instance, one study found a significant positive correlation between CTI and depressive symptoms, suggesting that CTI may serve as a potential clinical marker for identifying and stratifying depressive symptoms [11]. Additionally, CTI has been used to predict the risk of erectile dysfunction, with research indicating that men with higher CTI levels have a significantly increased risk of developing erectile dysfunction [12]. CTI is also closely associated with CVD. Studies have shown that CTI is positively correlated with the risk of coronary artery disease, and incorporating CTI significantly improves the ability to identify coronary artery disease [13]. CTI has also been found to be associated with stroke risk, with increased CTI levels correlating linearly with an increased risk of stroke in hypertensive patients [14].

This study utilizes a large-scale national cohort, the China Health and Retirement Longitudinal Study (CHARLS), conducted over a period from 2011 to 2020, enabling us to assess the long-term impact of CTI on CVD risk. To our knowledge, this study is among the first to apply restricted cubic spline (RCS) analysis to explore the nonlinear relationship between CTI and CVD, offering a more nuanced understanding of the association. Both indicators together may more comprehensively reflect the impact of metabolic disorders and inflammatory responses on cardiovascular health. Therefore, this study utilizes data from CHARLS conducted between 2011 and 2020 to explore the association between CTI and self-reported CVD, aiming to provide new perspectives and evidence for the early screening and prevention of CVD.

## 2 Methods

### 2.1 Study population and design

This study is based on the CHARLS, an ongoing nationwide prospective cohort study in China. CHARLS employs a proportional probability sampling method to collect nationally representative health-related data from the Chinese middle-aged and elderly population. The survey covers 28 provinces, 150 county-level units, and 450 village-level units, with the baseline survey involving approximately 10,000 households and over 17,000 individuals. The survey collects data on demographic background, health status and function, social and economic conditions, as well as retirement information [15].

**Fig 1:**
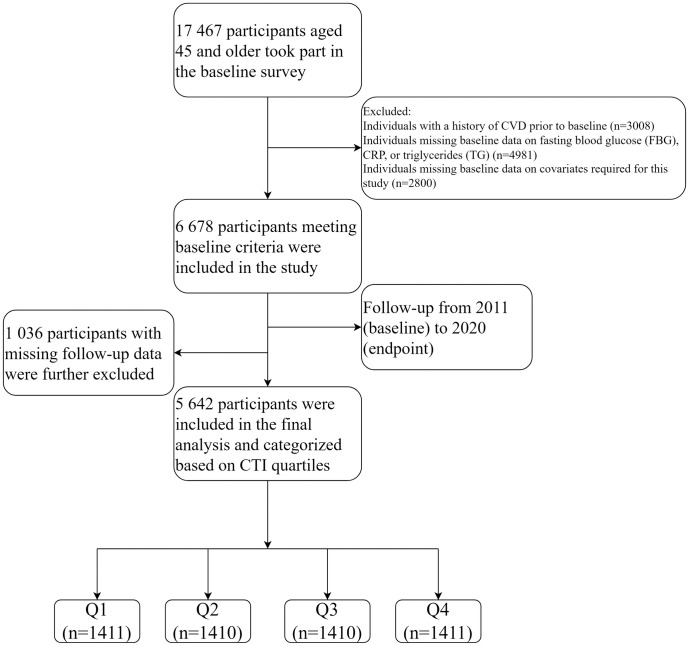
Flowchart illustrating the selection of the study population.

The current study involves a secondary analysis of the CHARLS data, including baseline data from 2011, and follow-up data from 2015, 2018, and 2020. Exclusion criteria included individuals with a history of CVD at baseline (i.e., heart disease or stroke), those missing complete data on fasting blood glucose (FBG), CRP, and triglycerides (TG), individuals with missing information on key variables, and those with missing CVD follow-up records from baseline to the 2020 endpoint. Missing data were handled using listwise deletion, and appropriate adjustments were made for the complex survey design, including the use of sampling weights and primary sampling units (PSUs) to ensure correct standard errors and valid statistical inferences. Ultimately, 5,642 participants were included in the final analysis. The flow chart of population selection in this study is shown in Fig 1. The data used in this study were approved by the Biomedical Ethics Review Committee of Peking University. Written informed consent was obtained from all participants. The ethical approval number is IRB00001052–11015.

### 2.2 Measurement of CTI

The CTI was defined as 0.412 × Ln [CRP (mg/L)] + Ln [TG (mg/dL) × FPG (mg/dL)/2]. In contrast to previous studies [12], the unit for CRP has been adjusted in this study to align with our specific measurement approach.

### 2.3 Assessment of CVD events

Assessment of CVD events includes heart disease and stroke [16]. Similar to previous studies, CVD events were assessed through the following questions: ‘Has a doctor ever told you that you have had a heart attack, angina, coronary heart disease, heart failure, or other heart problems?’ or ‘Has a doctor ever told you that you have had a stroke?’ Participants reporting heart disease or stroke were classified as having CVD.

### 2.4 Covariates

The selection of covariates was based on existing literature and theoretical considerations, with a focus on factors known to be associated with CVD. These covariates were carefully chosen to control for potential confounding and to ensure the robustness of the results. Demographic characteristics included age, gender, marital status, and residential area. Health-related factors included alcohol consumption, smoking status, body mass index (BMI), heart rate (PR), comorbidities (hypertension and diabetes), and laboratory indicators. Comorbidities, including hypertension and diabetes, were assessed based on self-reported diagnosis, where participants were asked whether a doctor had ever diagnosed them with these conditions. For laboratory indicators, we checked the distribution of each biomarker and adjusted for extreme values. Specifically, we set the lower and upper limits of each biomarker to the 1st and 99th percentiles, respectively, to minimize the impact of outliers on the analysis.

### 2.5 Statistical analyses

All analyses were performed using R statistical software version 4.2.2 (http://www.R-project.org, The R Foundation). Continuous variables are presented as medians with interquartile ranges (IQRs), while categorical variables are expressed as frequencies and percentages. The Kruskal-Wallis H test or chi-square test was used to compare continuous or categorical variables across different CTI quartiles.

Multivariable Cox proportional hazards regression models were employed to analyze the association between CTI and CVD risk, estimating hazard ratios (HRs) and their 95% confidence intervals (CIs). Model 1 was unadjusted for covariates. Model 2 adjusted for age, gender, marital status, residential area, alcohol consumption, smoking status, hypertension, and diabetes. Model 3 adjusted for age, gender, BMI, marital status, residential area, alcohol consumption, smoking status, hypertension, diabetes, pulse rate (PR), white blood cell count (WBC), hemoglobin (Hb), platelet count (PLT), hemoglobin A1c (HbA1c), uric acid (UA), blood urea nitrogen (BUN), creatinine (SCr), cystatin C (CysC), total cholesterol (TC), high-density lipoprotein cholesterol (HDL-C), and low-density lipoprotein cholesterol (LDL-C). In this study, CTI was modeled both as a continuous variable and as a categorical variable. Specifically, CTI was analyzed as a continuous variable, and three models were constructed, with HR representing the risk per unit increase in CTI. As a categorical variable, CTI was converted into quartiles, and three models were also constructed, with HR representing the risk of each quartile compared to the reference group.

To investigate the dose-response relationship between CTI and CVD, multivariable-adjusted restricted cubic spline (RCS) regression analysis was conducted. Furthermore, stratified analyses were performed based on age (<60 and ≥60), gender (female and male), BMI (<28 and ≥28), smoking status (never and past or current), alcohol consumption (never and past or current), diabetes (no and yes), hypertension (no and yes), and WBC (median, < 6.00 and ≥6.00). A two-sided *P* < 0.05 was considered statistically significant in this study.

## 3 Results

### 3.1 Baseline characteristics of study participants

**Fig 2:**
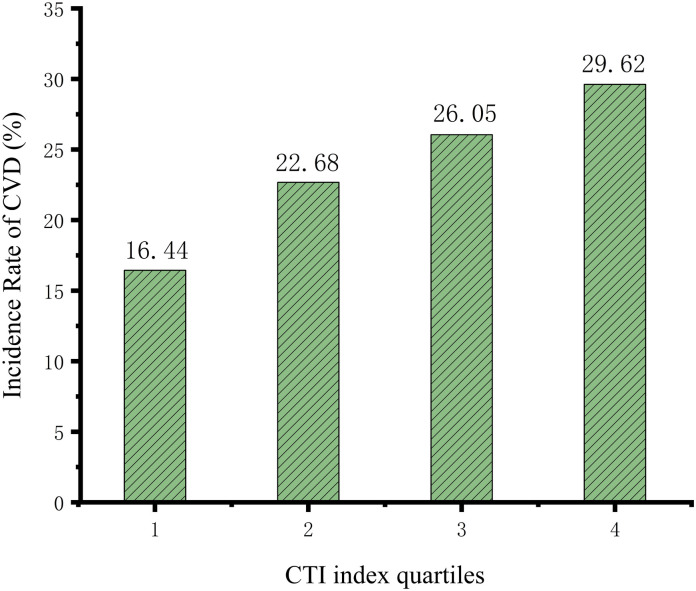
Bar chart of CVD incidence by CTI quartile groups.

**Fig 3:**
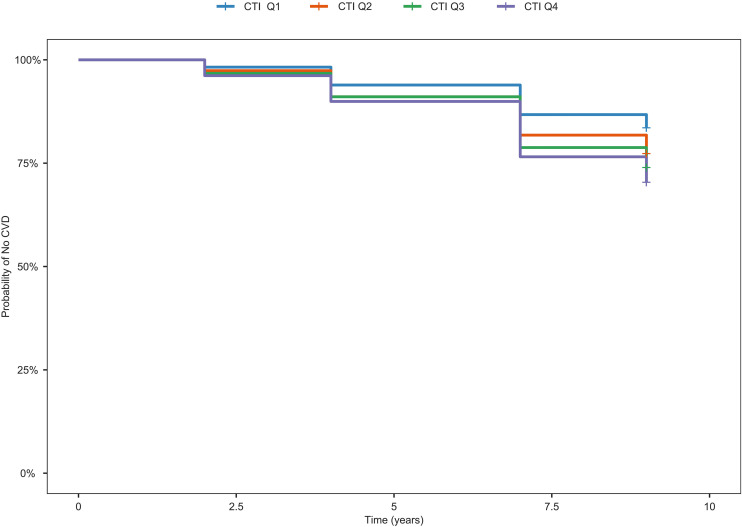
Kaplan-Meier curve for CVD risk by CTI quartile groups.

A total of 5,642 participants were included in the study. According to the CTI quartiles, the CVD incidence rates for these participants were 16.44%, 22.68%, 26.05%, and 29.62%, respectively (Fig 2). As the CTI quartile increased, the CVD incidence progressively increased, indicating a positive correlation between CTI and CVD risk. The Kaplan-Meier curve (Fig 3) further supports this finding, showing that participants with higher CTI values had a higher risk of developing CVD (*P*_Q1-Q2_ < 0.001; *P*_Q1-Q3_ < 0.001; *P*_Q1-Q4 _< 0.001; *P*_Q2-Q3 _= 0.044; *P*_Q2-Q4_ < 0.001; *P*_Q3-Q4_ = 0.037).

Additionally, the baseline characteristics of participants according to CTI quartiles are shown in Table 1. As the CTI quartile increases, several clinical characteristics show significant changes, particularly in hypertension, diabetes, and CRP levels. Specifically, the prevalence of hypertension significantly increases with higher CTI, rising from 14.32% in Q1 to 32.96% in Q4 (*P* < 0.001). The prevalence of diabetes also gradually increases with higher CTI quartiles, from 1.70% in Q1 to 10.84% in Q4 (*P* < 0.001). CRP levels significantly increase in higher CTI quartiles, with Q4 showing a CRP of 2.60 mg/L (1.38–5.49), significantly higher than Q1 at 0.49 mg/L (0.34–0.72) (*P* < 0.001). These results suggest that individuals with higher CTI quartiles may be at greater cardiovascular risk, particularly in terms of hypertension, diabetes, and inflammatory responses such as CRP, which warrants further attention.

**Table 1:** Baseline characteristics of participants by CTI quartile groups.

Variable	Overall	Q1	Q2	Q3	Q4	*P-*value
Age, years	59.00 (53.00 - 68.00)	59.00 (53.00 - 68.00)	58.00 (52.00 - 65.00)	59.00 (53.00 - 68.00)	59.00 (53.00 - 68.00)	<0.001
Male, n	2,696.00 (47.78%)	722.00 (51.17%)	701.00 (49.68%)	656.00 (46.56%)	617.00 (43.73%)	<0.001
BMI, kg/m^2^	22.89 (20.60 - 25.49)	22.86 (20.54 - 25.42)	22.89 (20.64 - 25.45)	22.97 (20.70 - 25.51)	22.82 (20.56 - 25.57)	0.813
Marital status, n						0.306
Other	755.00 (13.38%)	171.00 (12.12%)	194.00 (13.75%)	204.00 (14.48%)	186.00 (13.18%)	
Marred	4,887.00 (86.62%)	1,240.00 (87.88%)	1,217.00 (86.25%)	1,205.00 (85.52%)	1,225.00 (86.82%)	
Residential area, n						<0.001
Urban	1,868.00 (33.11%)	384.00 (27.21%)	437.00 (30.97%)	489.00 (34.71%)	558.00 (39.55%)	
Rural	3,774.00 (66.89%)	1,027.00 (72.79%)	974.00 (69.03%)	920.00 (65.29%)	853.00 (60.45%)	
Alcohol consumption, n						0.039
Never	3,384.00 (59.98%)	808.00 (57.26%)	835.00 (59.18%)	869.00 (61.67%)	872.00 (61.80%)	
Past or current	2,258.00 (40.02%)	603.00 (42.74%)	576.00 (40.82%)	540.00 (38.33%)	539.00 (38.20%)	
Smoking status, n						0.120
Never	3,387.00 (60.03%)	828.00 (58.68%)	824.00 (58.40%)	857.00 (60.82%)	878.00 (62.23%)	
Past or current	2,255.00 (39.97%)	583.00 (41.32%)	587.00 (41.60%)	552.00 (39.18%)	533.00 (37.77%)	
Hypertension, n	1,306.00 (23.15%)	202.00 (14.32%)	261.00 (18.50%)	378.00 (26.83%)	465.00 (32.96%)	<0.001
Diabetes, n	286.00 (5.07%)	24.00 (1.70%)	39.00 (2.76%)	70.00 (4.97%)	153.00 (10.84%)	<0.001
PR, bpm	72.00 (65.00 - 79.00)	72.00 (66.00 - 79.00)	72.00 (65.00 - 79.00)	72.00 (66.00 - 79.00)	72.00 (65.00 - 79.00)	0.551
CRP, mg/L	1.01 (0.54 - 2.13)	0.49 (0.34 - 0.72)	0.81 (0.53 - 1.33)	1.33 (0.78 - 2.41)	2.60 (1.38 - 5.49)	<0.001
WBC, × 10⁹/L	6.00 (4.95 - 7.20)	5.70 (4.70 - 6.90)	6.00 (5.00 - 7.40)	6.10 (5.10 - 7.30)	6.10 (5.00 - 7.23)	<0.001
Hb, g/L	14.20 (13.00 - 15.50)	13.90 (12.70 - 15.30)	14.20 (12.90 - 15.40)	14.20 (13.10 - 15.50)	14.40 (13.20 - 15.60)	<0.001
PLT, × 10⁹/L	207.00 (162.00 - 255.00)	204.00 (162.00 - 252.00)	202.00 (159.00 - 249.00)	207.00 (161.00 - 257.00)	217.00 (168.00 - 265.00)	<0.001
FBG, mg/dL	102.06 (94.14 - 112.68)	96.12 (89.10 - 103.50)	99.90 (92.88 - 107.82)	103.68 (95.76 - 113.04)	112.32 (101.52 - 134.46)	<0.001
HbA1c, %	5.10 (4.90 - 5.40)	5.00 (4.80 - 5.30)	5.10 (4.90 - 5.40)	5.10 (4.90 - 5.40)	5.30 (5.00 - 5.70)	<0.001
UA, mg/dL	4.29 (3.56 - 5.14)	4.00 (3.35 - 4.77)	4.21 (3.52 - 5.04)	4.36 (3.65 - 5.21)	4.63 (3.83 - 5.54)	<0.001
BUN, mg/dL	15.24 (12.60 - 18.21)	15.66 (12.74 - 18.77)	15.35 (12.63 - 18.37)	15.15 (12.58 - 18.12)	14.87 (12.49 - 17.53)	<0.001
SCr, mg/dL	0.76 (0.64 - 0.88)	0.75 (0.63 - 0.86)	0.75 (0.64 - 0.88)	0.76 (0.66 - 0.88)	0.77 (0.66 - 0.90)	<0.001
CysC, mg/L	0.98 (0.86 - 1.12)	0.96 (0.85 - 1.09)	0.99 (0.88 - 1.13)	0.99 (0.87 - 1.15)	0.97 (0.83 - 1.15)	<0.001
TC, mg/dL	190.21 (167.40 - 215.34)	181.70 (159.67 - 203.74)	187.50 (165.85 - 210.31)	194.46 (170.10 - 219.98)	200.26 (174.74 - 229.25)	<0.001
TG, mg/dL	103.54 (74.34 - 149.57)	65.49 (53.99 - 80.54)	94.69 (76.11 - 113.28)	123.01 (97.35 - 153.10)	184.08 (133.63 - 251.34)	<0.001
HDL-C, mg/dL	49.87 (40.98 - 60.31)	59.15 (49.87 - 69.20)	52.58 (45.23 - 61.86)	47.94 (40.59 - 56.83)	40.59 (33.63 - 48.71)	<0.001
LDL-C, mg/dL	114.43 (93.56 - 138.02)	108.25 (91.24 - 127.58)	115.21 (95.10 - 136.47)	120.23 (98.20 - 143.82)	114.82 (89.69 - 143.43)	<0.001
CTI	8.65 (8.14 - 9.21)	7.84 (7.61 - 8.00)	8.40 (8.27 - 8.53)	8.91 (8.78 - 9.05)	9.67 (9.42 - 10.03)	<0.001

Abbreviations: BMI, Body Mass Index; PR, Pulse Rate; CRP, C-Reactive Protein; WBC, White Blood Cell; Hb, Hemoglobin; PLT, Platelet; FPG, Fasting Blood Glucose; HbA1c, Hemoglobin A1c; UA, Uric Acid; BUN, Blood Urea Nitrogen; SCr, Serum Creatinine; CysC, Cystatin C;TC, Total Cholesterol; TG, Triglycerides; HDL-C, High-Density Lipoprotein Cholesterol; LDL-C, Low-Density Lipoprotein Cholesterol; CTI, C-reactive protein-triglyceride-glucose index.

### 3.2 Association between CTI and CVD

Table 2 shows the results of the multivariable Cox regression analysis. In the unadjusted model, CTI was significantly associated with an increased risk of CVD (HR = 1.33, 95% CI = 1.24–1.41). After multivariable adjustment, the results remained robust and statistically significant. Model 2 showed an HR of 1.21 (95% CI = 1.32–1.30, *P* < 0.001), and Model 3 showed an HR of 1.11 (95% CI = 1.01–1.23, *P* = 0.036). Furthermore, compared to the first CTI quartile, patients in the fourth quartile had a higher risk. Specifically, in Model 1, the HR for the Q4 group was 1.94 (95% CI = 1.65–2.28, *P* < 0.001); in Model 2, the HR was 1.61 (95% CI = 1.36–1.89, *P* < 0.001); and in Model 3, the HR was 1.33 (95% CI = 1.08–1.64, *P* = 0.008).

**Table 2:** Association between CTI and CVD.

	Model 1 HR (95% CI); *P-*value	Model 2 HR (95% CI); *P-*value	Model 3 HR (95% CI); *P-*value
CTI (per SD)	1.33(1.24-1.41); < 0.001	1.21(1.32-1.30); < 0.001	1.11(1.01-1.23); 0.036
CTI (quartiles)			
Q 1	(Reference)	(Reference)	(Reference)
Q 2	1.43(1.21-1.70); < 0.001	1.35(1.14-1.60);0.001	1.28(1.07-1.52); 0.006
Q 3	1.68(1.42-1.98); < 0.001	1.47(1.24-1.73); < 0.001	1.33(1.11-1.59); 0.002
Q 4	1.94(1.65-2.28); < 0.001	1.61(1.36-1.89); < 0.001	1.33(1.08-1.64); 0.008

Model 1 was unadjusted for covariates. Model 2 adjusted for age, gender, marital status, residential area, alcohol consumption, smoking status, hypertension, and diabetes. Model 3 adjusted for age, gender, BMI, marital status, residential area, alcohol consumption, smoking status, hypertension, diabetes, PR, WBC, Hb, PLT, HbA1c, UA, BUN, SCr, CysC, TC, HDL-C, and LDL-C.

Abbreviations: HR, Hazard Ratio; CI, Confidence Interval; SD, Standard Deviation; CTI, C-reactive protein-triglyceride-glucose index.

**Fig 4:**
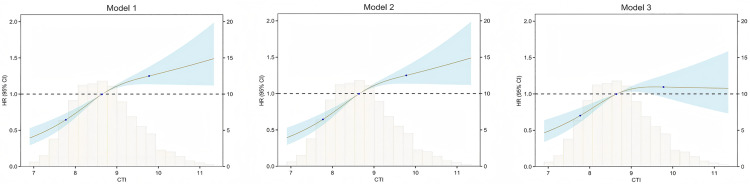
The non-linear relationship between CTI and CVD. Model 1 was unadjusted for covariates. Model 2 adjusted for age, gender, marital status, residential area, alcohol consumption, smoking status, hypertension, and diabetes. Model 3 adjusted for age, gender, BMI, marital status, residential area, alcohol consumption, smoking status, hypertension, diabetes, PR, WBC, Hb, PLT, HbA1c, UA, BUN, SCr, CysC, TC, HDL-C, and LDL-C.

Fig 4 illustrates the dose-response relationship between CTI and CVD. After adjusting for potential covariates, the nonlinear association between CTI and CVD remained statistically significant (*P* < 0.05).

### 3.3 Subgroup analysis of the association between CTI and CVD

**Fig 5:**
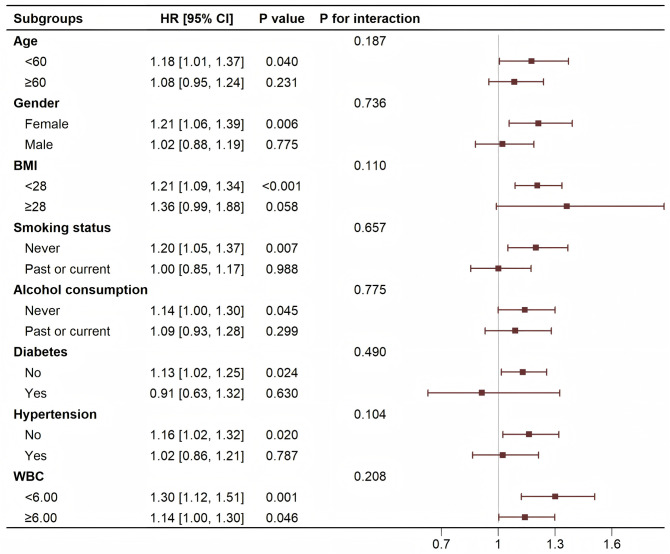
Association between CTI, and CVD in different subgroups. Abbreviations: HR, Hazard Ratio; CI, Confidence Interval; BMI, Body Mass Index; WBC, White Blood Cell.

After adjusting for covariates, subgroup analyses were performed based on age, gender, BMI, smoking status, alcohol consumption, diabetes, hypertension, and WBC. No significant interaction was found between CTI and CVD risk in any subgroup (all *P* for interaction > 0.05). Fig 5 presents the results of the CTI subgroup analysis, revealing a more significant association between CTI and CVD risk in patients with age < 60 years, female, BMI < 28, never alcohol consumption, never smoking status, no diabetes, no hypertension, and WBC.

## 4 Discussion

CVD is closely linked to inflammation and IR. Inflammation plays a key role in atherosclerosis, the main cause of CVD, by promoting the formation of atherosclerotic plaques, which can lead to ischemic events like myocardial infarction and stroke. Recent studies highlight the role of the NOD-like receptor family pyrin domain containing 3 (NLRP3) inflammasome in atherosclerosis and its progression to CVD [17,18]. Inflammation is not limited to the arterial walls but involves systemic interactions, as shown by leukocyte involvement in ischemic CVD [19]. Additionally, inflammation interacts with other risk factors like oxidative stress, further contributing to atherosclerosis and CVD [20]. Given its central role, anti-inflammatory therapies could provide benefits in preventing cardiovascular events and improving patient outcomes [21].

Several studies have underscored the vital role of CRP in the pathogenesis of CVD. For example, a meta-analysis of systematic reviews and randomized controlled trials highlighted the effectiveness of statins in lowering CRP levels, which in turn was associated with a reduced risk of cardiovascular events in CVD patients [22]. The Dallas Heart Study found that the association between CRP and atherosclerosis was influenced by BMI, with a more significant relationship observed in non-obese individuals [23]. In a population-based survey, an ideal cardiovascular health score was negatively correlated with inflammation, as indicated by CRP and white blood cell count, further emphasizing the importance of maintaining a healthy lifestyle to reduce cardiovascular risk [24]. Additionally, the ARIC study indicated that inflammation was independently associated with atherosclerotic cardiovascular disease (ASCVD) regardless of atherosclerotic lipid levels [25]. These studies collectively highlight the importance of CRP as a biomarker for CVD risk and underscore its potential role in predicting cardiovascular events and guiding therapeutic interventions in both clinical and research settings.

IR is another critical factor in the pathogenesis of CVD. IR leads to endothelial dysfunction, which is a precursor to the formation of atherosclerotic plaques. This dysfunction manifests as reduced nitric oxide bioavailability, increased oxidative stress, and enhanced inflammation, all of which accelerate the progression of atherosclerosis [26,27]. Atherosclerotic dyslipidemia, often associated with IR, is characterized by elevated TG, low HDL-C, and the presence of small, dense low-density lipoprotein particles. These lipid abnormalities play a key role in the occurrence and development of atherosclerotic lesions [28]. The lipid metabolism dysregulation in IR states leads to increased lipid deposition in the arterial walls, promoting the formation of atherosclerotic plaques [29]. Furthermore, IR is closely associated with systemic inflammation, which further leads to endothelial dysfunction and atherosclerosis. In individuals with IR, the inflammatory response is typically characterized by elevated levels of pro-inflammatory cytokines such as TNF-α and IL-6, which are known to impair insulin signaling and exacerbate vascular inflammation [30,31]. The relationship between IR and atherosclerosis is also influenced by oxidative stress, which is both a cause and a consequence of endothelial dysfunction. Reactive oxygen species generated in IR states can damage endothelial cells, further impair vascular function and promote the formation of atherosclerotic plaques [32,33].

The TyG index, as an alternative marker for IR, is closely associated with the risk of cardiovascular disease. Studies have shown that in patients with familial hypercholesterolemia, an elevated TyG index is significantly associated with an increased risk of ASCVD, with a 74% increase in ASCVD risk for every unit increase in the TyG index [34]. In non-diabetic individuals, the TyG index has also been found to be significantly associated with the risk of congestive heart failure, stroke, and hypertension [35]. Moreover, the TyG index has been shown to be positively linearly correlated with major adverse cardiovascular events (MACE) in patients with coronary artery disease, with a significant increase in MACE risk when the TyG index reaches or exceeds 8.73 [36]. The TyG index, as a simple and practical indicator, can effectively predict the risk of CVD and has demonstrated its significant clinical value in various populations.

An elevated CTI not only reflects increased inflammation but also indicates worsening IR, both of which play critical roles in the pathogenesis of CVD. This study, based on data from the CHARLS conducted between 2011 and 2020, explored the association between CTI and CVD risk. To our knowledge, this study is among the first to apply RCS analysis to explore the nonlinear relationship between CTI and CVD, offering a more nuanced understanding of the association. The results revealed a significant positive correlation between CTI and CVD risk, supporting CTI as a comprehensive indicator capable of effectively reflecting the impact of inflammation and IR on cardiovascular health. Specifically, for every unit increase in CTI, the risk of CVD increased by 11% (HR = 1.11, 95% CI = 1.01–1.23). Moreover, RCS analyses further revealed a nonlinear relationship between CTI and CVD. Another key finding of this study was the heterogeneity in the association between CTI and CVD. Subgroup analysis showed that the relationship between CTI and CVD risk was less significant in certain specific populations compared to the overall sample, suggesting that the relationship between CTI and CVD may be influenced by demographic characteristics, lifestyle factors, and other metabolic factors. These factors may differ in their impact on inflammation and metabolic disturbances across different groups, thereby affecting the role of CTI in CVD prediction. By providing a comprehensive evaluation of CTI, clinicians may gain a more holistic predictive tool for assessing patients’ cardiovascular health and early identification of high-risk populations, ultimately helping to implement more effective prevention and intervention strategies.

## 5 Study limitations

Our study has several limitations that should be acknowledged. First, despite adjusting for a variety of covariates, there may still be unmeasured potential covariates that could affect the relationship between CTI and CVD. Model 3 included many variables, some of which may act as mediators in the causal chain, and adjusting for these variables could distort the causal relationship. Future studies should be more cautious in considering the role of these variables, particularly their potential as mediators rather than merely covariates. Second, the data were derived from self-reported questionnaires, which may introduce recall bias or inaccuracies in reporting, potentially affecting the reliability of the results. Specifically, previous studies have shown that the reporting rate of chronic diseases (including CVD) in the CHARLS data is relatively high, meaning that some participants may not accurately report their CVD history, leading to an underestimation of CVD risk. Moreover, as CRP is an acute-phase reactant produced by the liver and reflects short-term inflammation, its variability due to acute inflammation should be considered a limitation when interpreting CTI. High CRP levels may not necessarily indicate chronic inflammation, and some participants with elevated CRP may reflect transient inflammation rather than long-term inflammatory processes, which could affect the robustness of the findings.Third, as CHARLS is conducted only in China, caution is needed when generalizing our findings to other populations. Therefore, additional studies involving diverse populations are necessary to validate our results. Fourth, although we used a cohort design, future studies should further verify the causal relationship between CTI and CVD and expand the sample size to ensure the broader applicability of the results. Fifth, in handling missing data, we employed listwise deletion. While this method is commonly used, it does not address potential biases caused by non-random attrition. To minimize the impact of attrition bias, we recommend that future studies consider using inverse probability weighting (IPW) or other methods to account for baseline characteristics that influence participation in follow-up, which could improve the accuracy of the findings. Finally, we adjusted for extreme values in laboratory biomarkers by setting the lower and upper limits to the 1st and 99th percentiles, respectively, to minimize the influence of outliers on the results. However, the impact of extreme values may not be completely eliminated, and future studies may explore other methods to handle these values.

## 6 Conclusion

Higher CTI is significantly associated with an increased risk of CVD. This study supports the utility of CTI as a valuable biomarker that reflects both inflammation and insulin resistance—two key factors in the pathogenesis of CVD.

Given the central role of inflammation and insulin resistance in CVD development, CTI could potentially serve as an early predictor of CVD risk. Additionally, by combining multiple biomarkers into a single index, CTI offers a more holistic measure of cardiovascular risk compared to individual markers. Future studies should further explore the clinical applications of CTI, evaluate its performance in different populations, and investigate its potential as a target for therapeutic interventions aimed at reducing cardiovascular events.
